# Transplantation for renal failure secondary to enteric hyperoxaluria: a case report

**DOI:** 10.1186/1752-1947-1-31

**Published:** 2007-06-25

**Authors:** Stephen I Rifkin

**Affiliations:** 1 Division of Nephrology, University of South Florida College of Medicine, 2403 W. Azeele St. Tampa, Florida, 33609, USA

## Abstract

Enteric hyperoxaluria can lead to renal failure. There have only been a few reports of renal transplantation as treatment of endstage renal disease secondary to enteric hyperoxaluria and results have been mixed. This report describes a patient with Crohn's disease who developed chronic renal failure from enteric hyperoxaluria. He subsequently had a successful renal transplant without any post-operative oxalate related complications and has satisfactory renal function almost three years later. Aggressive pre-transplant hemodialysis was not done. The literature associated with renal transplantation for enteric hyperoxaluria is reviewed.

## Background

Enteric hyperoxaluria may occur in patients with intestinal malabsorption from a variety of causes. Complications include oxalate stone disease, acute renal failure, and oxalate induced interstitial nephritis with the development of chronic renal insufficiency. There have been rare reports of renal transplantation for the resulting end stage renal disease and these reports have often been complicated by oxalate deposition and renal insufficiency or graft loss [1-5]. This report is of a patient with longstanding Crohn's disease, short bowel syndrome from surgery with resultant hyperoxaluria and renal failure secondary to recurrent stone disease. He underwent successful deceased donor renal transplantation without any post-op oxalate related complications and with satisfactory renal function almost three years post transplant.

## Case Presentation

The patient is a 60 year old white male who had Crohn's disease diagnosed in the 1960's. He had small bowel resections in 1965 and 1969. He developed recurrent stone disease in the early 1980's. In 1999 24 hour urine studies showed an elevated oxalate excretion of 1.29 mmol (normal <0.50 mmol) and low magnesium, citrate, and calcium excretion. His serum creatinine was 2.0 mg% in 1995 and 2.5 mg% in Nov. 2001. In June, 2002 he presented with acute renal failure secondary to obstructive stone disease with a serum creatinine of 12.7 mg%. With correction of the obstruction his renal function only modestly improved and he was placed on hemodialysis on 7/29/02. His dialysis course was complicated by multiple blood access problems and several episodes of life-threatening sepsis. As a result he underwent deceased donor renal transplant on 3/9/04. Induction therapy consisted of basiliximab, mycophenolate mofetil, and steroids and then maintenance therapy with tacrolimus, sirolimus, and steroids was instituted. The patient did not undergo intensive hemodialysis either prior to or after transplantation. He did have an acute rejection episode. The biopsy showed Banff 1b acute rejection, but no evidence of oxalate deposition. 24 hour urinary oxalate excretion was elevated at 113.3 mg (normal = 3.6–38 mg/24 hr). His rejection episode was treated with antithymocyte globulin (rabbit) with an excellent response. He was discharged on calcium with meals, Urocit-K, a low oxalate diet, and pyridoxine. His post-hospital course has been uneventful and his serum creatinine is 1.5 mg% almost three years later.

## Discussion

Under normal circumstances ingested calcium binds with oxalate in the intestines to form an insoluble complex. With extensive intestinal bypass or short gut situations malabsorption and steatorrhea cause intraluminal calcium to bind preferentially with bile salts. Thus, more oxalate is absorbed. In addition, colonic absorption of oxalate increases due to mucosal alterations brought about by the entry of malabsorbed fatty acids and bile salts into the colon. Other possible contributing factors include metabolic acidosis, a concentrated urine from chronic diarrhea, and reduced urinary concentration of magnesium and citrate [6].

Oxalate deposition can lead to an interstitial inflammatory response and interstitial fibrosis. This appears to be caused by a variety of toxic responses in renal epithelial cells to oxalate exposure including altered membrane surface properties, changes in gene expression, disruption of mitochondrial function, formation of reactive oxygen species, activation of phospholipase A2, upregulation of cyclooxygenase-2, and decreased cell viability [7]. Increased synthesis of osteopontin, bikunin, heparan sulfate, monocyte chemoattractant protein 1, and prostaglandin E2 which are known to participate in inflammatory processes and in extracellular matrix production has also been noted [8].

Treatment of hyperoxaluria could include oral calcium supplements given with meals to bind intestinal oxalate, cholestyramine to bind bile salts and fatty acids, increased oral fluids, citrate administration, a low oxalate, high calcium, low fat diet, use of an organic marine hydrocolloid that helps adsorb oxalate within the gut lumen, colonic degradation of endogenous oxalate by orally administered Oxalobacter formigenes, and treatment of the primary cause such as converting a jejunal-ileal bypass to a roux-en-y bypass.

The plasma oxalate level increases starting at a glomerular filtration rate of about 30 ml/min and oxalate retention increases rapidly when the glomerular filtration rate decreases below about 20 ml/min [7]. In otherwise normal dialysis patients serum oxalate levels remain elevated even though dialysis removes significant mounts of oxalate [8]. However, significant organ dysfunction does not generally occur in the dialysis population [9]. In addition, substantial oxalate deposition post transplant generally does not occur [10].

There have only been a few reports of renal transplantation in patients with endstage renal disease secondary to enteric hyperoxaluria. Results have been mixed. Roberts et al [1] reported a patient who had stable renal function (serum creatinine of 120 mmol/L) 10 months after transplant inspite of having to be treated for an acute rejection episode on day 21. A renal biopsy at that time did not show any oxalate deposition. No further followup is given. Cuvelier et al [2] reported a patient who had two successive renal transplants 7 months apart. Both grafts showed widespread oxalate deposition on early biopsies and neither graft initially functioned. The second graft's function improved sufficiently 11 months after transplant to allow discontinuation of dialysis. Approximately 4 years later serum creatinine was 3.0 mg%. Kistler et al [3] reported a patient who had a creatinine clearance of 60–70 ml/min seven years after transplant inspite of the demonstration of oxalate crystal deposition on day 9 after transplant. This patient underwent intensive hemodialysis after the transplant. Bernhardt et al [4] report a patient who received daily hemodiafiltration (3 hours) for two weeks after transplant. Biopsy on day 11 showed borderline rejection as well as sporadic deposition of oxalate crystals. Unfortunately, serum creatinine one and a half years later was approximately 5 mg%. Lefaucheur [5] mention a patient with mucoviscidosis who developed acute oxalate-induced renal failure four months after a nonrenal organ transplant. He had a renal transplant 3 years later with a post-transplant serum creatinine of 1 mg/dL, but no other details are given.

The results of renal transplantation alone for the treatment of renal failure secondary to primary hyperoxaluria type I, a disease with substantial overproduction of oxalate, are generally not adequate. The addition of liver transplantation, which corrects the enzyme deficiency, produces better results [13]. This suggests that the present patient will require continued monitoring and treatment of his enteric hyperoxaluria.

## Conclusion

Renal transplantation for chronic renal failure resulting from enteric hyperoxaluria is a reasonable treatment option. Aggressive pre-transplant dialysis may not be necessary.

## Competing interests

The author(s) declare that they have no competing interests.
